# Electrospray Ionization Efficiency Is Dependent on Different Molecular Descriptors with Respect to Solvent pH and Instrumental Configuration

**DOI:** 10.1371/journal.pone.0167502

**Published:** 2016-12-01

**Authors:** Andreas Kiontke, Ariana Oliveira-Birkmeier, Andreas Opitz, Claudia Birkemeyer

**Affiliations:** 1 Institute of Analytical Chemistry, University of Leipzig, Linnéstr., Leipzig, Germany; 2 Institute of Linguistics, University of Leipzig, Beethovenstr., Leipzig, Germany; University of Edinburgh, UNITED KINGDOM

## Abstract

Over the past decades, electrospray ionization for mass spectrometry (ESI-MS) has become one of the most commonly employed techniques in analytical chemistry, mainly due to its broad applicability to polar and semipolar compounds and the superior selectivity which is achieved in combination with high resolution separation techniques. However, responsiveness of an analytical method also determines its suitability for the quantitation of chemical compounds; and in electrospray ionization for mass spectrometry, it can vary significantly among different analytes with identical solution concentrations. Therefore, we investigated the ESI-response behavior of 56 nitrogen-containing compounds including aromatic amines and pyridines, two compound classes of high importance to both, synthetic organic chemistry as well as to pharmaceutical sciences. These compounds are increasingly analyzed employing ESI mass spectrometry detection due to their polar, basic character. Signal intensities of the peaks from the protonated molecular ion (MH^+^) were acquired under different conditions and related to compound properties such as basicity, polarity, volatility and molecular size exploring their quantitative impact on ionization efficiency. As a result, we found that though solution basicity of a compound is the main factor initially determining the ESI response of the protonated molecular ion, other factors such as polarity and vaporability become more important under acidic solvent conditions and may nearly outweigh the importance of basicity under these conditions. Moreover, we show that different molecular descriptors may become important when using different types of instruments for such investigations, a fact not detailed so far in the available literature.

## Introduction

Electrospray ionization for mass spectrometry (ESI-MS) is increasingly employed to analyze a broad range of target analytes from many compound classes, but is known to be particularly suitable for soluble, polar analytes. For selection of an appropriate analytical technique, responsiveness is one of the critical parameters for the quantitation of chemical compounds [1]. ESI response in particular can vary significantly among different analytes that have identical solution concentrations [2–6]; thus, different solute ionization efficiency can result in apparent responses that differ by > 3 orders of magnitude despite equimolar concentrations in solution [7]. Besides fundamental interests such as the suitability of ESI for the analysis of a particular analyte, data on molar responses can also provide general usefulness in analytical method development such as understanding the underlying mechanisms enabling the selection of optimal instrumental parameters, deducing molecular characteristics of an unknown species or information on the feasibility of calibration without chemical standards [7].

Chargeability of the analyte is of key importance for ionization in the electrospray process [8]. Ions can be formed by charge separation (e.g. deprotonation) or adduct formation (e.g. protonation) in the solution or gas phase [6], or by electrolytic oxidation or reduction [9,10]. The major steps of gas phase ion formation during the electrospray process are: (a) the production of charged droplets in the outlet of the capillary tip, (b) droplet disintegration to very small and highly charged droplets, due to evaporation of the solvent, and (c) the formation of gas phase ions. The transfer of ions to the gas phase is described (i) by the *Ion Evaporation Model* (IEM) [11], in which the increased charge density that results from solvent evaporation eventually causes Coulombic repulsion to overcome the liquid’s surface tension, resulting in a release of ions from the droplet surfaces, or (ii) by the *Charge Residue Model* (CRM) [12], where the increased charge density due to solvent evaporation causes large droplets to divide into smaller and smaller droplets, which finally consist only of single ions. Hence, ESI-MS response will depend on all analyte and solvent characteristics, and other parameters influencing the abovementioned processes.

In general, pioneers in the field of ESI-MS have always emphasized the importance of analyte physicochemical properties in describing ionization efficiency [3,12–14]. Despite many research efforts aiming at delineating the many factors that influence ESI-MS ionization efficiency of analytes, a comprehensive model still remains elusive largely due to the wide variability in analyte behavior under different conditions encountered in the ESI process. Moreover, several studies were done neglecting a potential effect of upstream applied separation techniques [7,15–17]. Most studies involved a limited number of compounds and of different characteristics so that only few systematic studies involving a reasonable number of compounds are available. Also, the investigated compounds often were polyfunctional and had a limited range of ionization efficiencies which hampered relating ionization efficiency successfully to the molecular structure [18]. Therefore, a reasonably large, fully characterized set of analytes was suggested to be ideal for the identification of general trends underlying the process [19].

Henriksen et al. [20] concluded that, due to the complexity of the ESI process, it was difficult to correlate the responsiveness of small molecules to ESI-MS with a single parameter. As a consequence, multivariate analyses are required broadening the scope of such investigations to many physicochemical properties and many compounds assessed at a time, extracting also the interactions between all assessed parameters [17–19,21–24]. Thus, Mandra et al. [23] investigated 84 factors of several pharmaceuticals and found the number of free rotatable bonds positively correlated to ESI-MS response in addition to the confirmation of known parameters such as pKb, logD (at high pH), the molecular volume related to the molecules polarizability, and the polar surface area. However, in this study the presence of other than protonated adducts was neglected possibly distorting and/or obscure potential relationships.

Thus, for a systematic investigation of the impact of compound characteristics and solvent pH on ESI-MS responsiveness we selected a number of 56 nitrogen-containing, aromatic compounds (58 in total). The compound group was not only chosen with respect to their importance in biological as well as in materials science [25,26], but also based on the reasoning that many compounds analyzed today by ESI contain structural units that are similar to our analytes. Moreover, these analytes and their features are not only well characterized in the literature and publicly available databases such as Scifinder, ChemAxon, and NIST Chemistry WebBook but they are also commercially available in a very broad structural variety.

Therefore, our experiment was designed to specifically investigate compound type-specific determinants of ESI-MS response. The selection of compounds was driven to overcome the limitations of many ESI models built on diverse classes of solutes that differ significantly in terms of their charge, polarity, or size [7]. To the best of our knowledge, there has been no earlier investigation reported on ESI-MS flow injection of such a large number of fully characterized compounds, carefully selected by their principal chemical structure for a systematic study of the influence of the compound characteristics such as basicity, polarity, vapor pressure, and molecular size on ESI-MS response. In addition, the adjustment of solvent pH and differential ion suppression effects occurring when using pH modifiers were investigated.

## Materials and Methods

### Chemicals

4-aminobenzene-sulfonic acid (sulfanilic acid) was purchased from Riedel-de-Haen AG (Hannover, Germany). 3-methoxyphenylhydrazine (hydrochloride, HCl) was purchased from Alfa Aesar (Karlsruhe, Germany), 2-methylaniline (*o*-toluidine), 4-methylaniline (*p*-toluidine) from Fluka (Buchs, Switzerland) and aniline from Acros (Geel, Belgium). Ammonium chloride was purchased from VWR (Dresden, Germany). Acetonitrile (ACN) ≥ 99.95%, LC-MS grade and formic acid (HCOOH) were purchased from Carl Roth (Karlsruhe, Germany) and water from BIOSOLVE (Valkenswaard, Netherlands). 4-nitro-1,2-phenylenediamine (hydrochloride, HCl), sodium chloride (NaCl), sodium fluoride (NaF), ammonium fluoride (NH_4_F), sodium formate and ammonium formate were purchased from Merck (Darmstadt, Germany). 2-aminoaniline (*o*-phenylenediamine), 2-aminopyridine, 2-aminophenol, 4-aminophenol, 2-aminobenzoic acid, 3-aminobenzoic acid, 4-aminobenzoic acid, and 4-chloroaniline were kindly provided by Prof. *emeritus* S. Berger (Institute of Analytical Chemistry, University of Leipzig, Germany). All other compounds were purchased from Sigma Aldrich (Taufkirchen, Germany). The detailed structures of all analytes are summarized in S1 Fig.

Before starting an experiment, the solvent pH was confirmed including the analyte solutions at the concentrations at which the analytes were dissolved. For all solutions, deionized water at pH 7 was used with careful degassing, due to the rapid pH decrease in the aqueous solutions shortly after degassing so that constant degassing and close pH control was prerequisite for all investigations. All solutions were freshly prepared before analysis.

Before starting the experiments, we confirmed that the selected analyte concentration was within the dynamic range.

### Assessment of relative basicity of the compounds

The pH of a 4 mM solution was determined with a pH electrode inoLab® pH Level 1 precision pH meter calibrated using pH calibration solutions at pH 4.0, pH 7.0 and pH 9.0 (all WTW Weilheim, Germany). Deionized water at pH 7 was used to prepare the compound solutions. Molarity of liquid analytes was calculated according to the density of the substance obtained from the Sigma Aldrich website. To the hydrochlorides or hemi sulfates (4,5-diamino-6-hydroxypyrimidine, 4-methoxyphenylhydrazine, 3-methoxyphenylhydrazine, 5,6-diamino-2,4-dihydroxypyrimidine and 4-hydrazinopyridine) 4 mM NaOH was added, 8 mM NaOH to the dihydrodichloride (4-methoxy-*o*-phenylenediamine) and the dihydrosulfate (6-hydroxy-2,4,5-triaminopyrimidine).

### Flow injection ESI-MS analyses

To keep solvent characteristics such as vapor pressure, surface tension and temperature constant, solvent composition was not changed within an experiment with the exception of the pH modifier for solvent acidification. ESI analyses were conducted as flow injection analyses in positive ion mode.

For the first experiment, 40 μM solutions of each analyte were prepared in 80% ACN or in 80% ACN with 0.02 M formic acid (pH 3). Each of the replicate solutions was introduced by a syringe pump at a flow rate of 3 μL/min. After each sample analysis a blank solution was injected (80% ACN and water) to equilibrate the system. Analyses were conducted on an API 2000 Triple Quadrupole equipped with the Analyst® 1.4.2 Software (AB Sciex, Toronto, Canada). The following instrumental parameters were used: Turbo Ion Spray voltage 5000 V, source gas 2 20 psi, dry gas temperature 50°C, focusing potential 200 V, entrance potential 10 V. The *m/z* range was set between 50 and 450 with a scan duration of 5 sec. After analysis of the declustering potential for all amino compounds, it was set to the optimum value of 60 V which was suitable for all analytes without signal loss for our eluent system. After the TIC reaching a constant intensity, each mass spectrum was acquired at least for 2 minutes and the average response of each analyte was calculated from triplicate analysis.

The set of anilines was reanalyzed twice using automated direct injection by an autosampler and pump of an HPLC system. For this, 10 μM solutions of each aniline were prepared in 50% ACN or in 50% ACN adjusted with formic acid to pH 3. First analysis was carried out using a JASCO 900 autosampler (JASCO, Gross-Umstadt, Germany) at a flow rate of 20 μL/min (lower instruments limit) and 100 μL sample volume. Mass spectra were acquired on the API 2000 instrument with the following instrumental parameters: Turbo Ion Spray voltage 5500 V, source gas 1 (GS1) and source gas 2 (GS2) were set to 25 and 70 psi respectively (both nitrogen), temperature 0, focusing potential 100 V, entrance potential 10 V, a declustering potential of 45 V for this eluent system. The *m/z* range was set between 65 and 250 with a scan duration of 3 sec. Each mass spectrum was acquired at least for 1 min after the signal intensity reached a constant value (12 min analysis time including subsequent equilibration). The average response of each analyte was calculated from triplicate analysis.

For the second analysis, a Bruker Esquire 3000+ ESI-ion trap MS operated by the Bruker esquire control 5.3 software and equipped with an Agilent 1100 autosampler and binary pump for automated flow injection in positive ESI-mode was used. The capillary voltage was at 4.5 kV, the target mass was set to *m/z* 120, 50 or 100 μL sample was injected at flow rate of 50 μL/min (lower instruments limit) with 70 psi nebulizer and 12 L/min dry gas flow rate (both nitrogen) at 320°C. These values were selected to minimize the intensity of the sodium adduct of nitroaniline. A maximum of 20,000 ions/ scan was collected in a scan range between *m/z* 50–250 at a maximum accumulation time of 200 ms and 3 scans rolling average resulting in a scan duration of ~0.4 sec. The *m/z* peak signal intensities were averaged over 1 min analysis time using Bruker Data analysis software 3.3 and the corresponding signal intensities of triplicate analyses were used for data evaluation.

For assessment of ion suppression, 10 μM solutions of each analyte at pH 3 adjusted by formic acid or HCl, respectively, or 1 mM solutions of sodium chloride, ammonium chloride, ammonium formate, or sodium formate in 50% ACN were measured in triplicate using flow injection from a syringe pump at a flow rate of 20 μL/min (lower instruments limit) with subsequent ESI-MS analysis on the API 2000. For confirmation, 10 μM solutions of each analyte were prepared in 50% or 80% ACN, respectively, or in 50% or 80% ACN at pH 3 adjusted by formic acid or HCl, respectively, or 1 mM solutions of sodium chloride, ammonium chloride, ammonium formate, sodium formate, sodium fluoride or ammonium fluoride and introduced in triplicate using flow injection from an Agilent HPLC pump at flow rate of 50 μL/min with subsequent ESI analysis on the Esquire 3000+.

### Data evaluation

The responsiveness of a given compound was assessed as the average intensity (cps, peak height) of the corresponding peak for the MH^+^ ion of the analyte of interest or, if present, as the sum of the molecular ion and its CID product, MH^+^ and MH^+^—NH_3_ (or MH^+^—H_2_O for 2-aminobenzoic acid) considering that the CID product likely originates from an earlier formed protonated molecule (mainly for the phenylenediamines and electron-rich hydrazines), and for chloroaniline as sum of the two most abundant isotopes. Response ratios pH3 / pH7 were calculated from the average values at different solvent pH. For the set of anilines, response of the substituted anilines was normalized to the response of aniline to enable comparisons of interday analyses.

Characteristic chemical constants (pKa, polar surface area, solvent accessible molecular surface area, logP, logD, proton affinity, gas phase basicity, boiling point, vapor pressure, vaporization enthalpy, surface tension) were obtained from public databases, namely ChemSpider by the Royal Society of Chemistry, London [http://www.chemspider.com/], chemicalize.org by ChemAxon, Budapest, Hungary [http://www.chemicalize.org/], Scifinder by the Chemical Abstracts Service, Columbus/Ohio, USA [https://scifinder.cas.org/], and the NIST Chemistry WebBook by The National Institute of Standards and Technology (NIST, Gaithersburg, USA) [http://webbook.nist.gov/chemistry/]. The molecular volume was calculated using the Spartan software package (Spartan 14, Wavefunction Inc., Irvine, USA). The settings for calculation were DFT (density functional theory) B3LYP with a 6–31G* basis set.

Peak signal intensities correlation analysis with all obtained physicochemical characteristics was carried out using linear (Pearson’s product-moment correlation coefficient) and non-linear correlation (Spearman’s rank correlation coefficient) in MS Excel 2013 (Microsoft Corp., Redmond, USA) and the “R” software version 3.3.1 (R Core Team, 2016: A language and environment for statistical computing. The R Foundation for Statistical Computing, Vienna, Austria) [https://www.R-project.org/]. All coefficients are listed in S1 Table. A scatterplot matrix was created in R 3.2.3 (The R Foundation for Statistical Computing 2015) using the “pairs” function to confirm appropriate data distribution before correlation analysis (S3 Fig).

## Results and Discussion

The systematics and chemical structures of the 58 compounds in this investigation comprising anilines, pyridines, hydrazines and pyrimidines with mostly one more substituent are illustrated in S1 Fig. We investigated the MH^+^ ESI responsiveness of this set of compounds independent on instrumental parameters, i.e. we kept the instrumental conditions constant during analysis. Also, we avoided any interaction such as charge competition between the analytes [27] introducing them separately at a low concentration. Finally, we kept the solvent composition, the retention in the instrumental system and the transient signal during the analysis constant using direct injection for all experiments instead of introducing the compounds after separation with, for instance, LC or CE [7,15–17]. The compounds we used were selected to overcome the limitations of other studies using diverse classes of solutes differing in charge, polarity, or size [7]: our analytes were all singly charged and featured molecular masses between 79 and 198 g/mol (119 g/mol on average, SD = 22 g/mol). For the anticipated set of analytes, namely aromatic, nitrogen-containing compounds, protonation plays the predominant role in ion formation, other ion adducts than the protonated ones, if observed at all, were detected only in negligible amounts.

### The fundamental relationship between ESI-MS response of the MH^+^ and solution basicity applies to the nitrogen-containing aromatic compounds

One of the most important compound characteristics known to determine the intensity of the MH^+^ signal in mass spectrometry after electrospray ionization is the extent of its protonation in solution, i.e. the solution basicity [23,28–32]. In agreement, our data reflects this fundamental relationship; however, since this correlation has been already extensively studied in the available literature (not for our analytes though) we shifted the detailed results and the discussion of the influence of solution basicity on relative signal intensity of our nitrogen-containing compounds to S1 Appendix. In summary, we found solution basicity so closely related with the interplay of electron-donating and -withdrawing effects in the structure of the analyte, i.e. the electron density of the investigated molecules, that these parameters cannot be separately assessed; ESI-response is determined to the same extent by solution basicity as by the structural effects that also account for the basicity of a compound.

### The extent of ion suppression or signal enhancement by pH modifiers is related to compound basicity and the type of the instrument

In analyses employing positive ion mode ESI-MS it is common practice to add a pH modifier to the solvent to improve signal intensities of target analytes [24,28,33–34]. Although the addition of formic acid to the eluent as ion pairing reagent is beneficial improving RP-HPLC performance of many analytes, contradictory results were reported whether or not a low pH is indeed beneficial for the ESI-response of protonated analytes [15,17,28,30,35].

**Fig 1:**
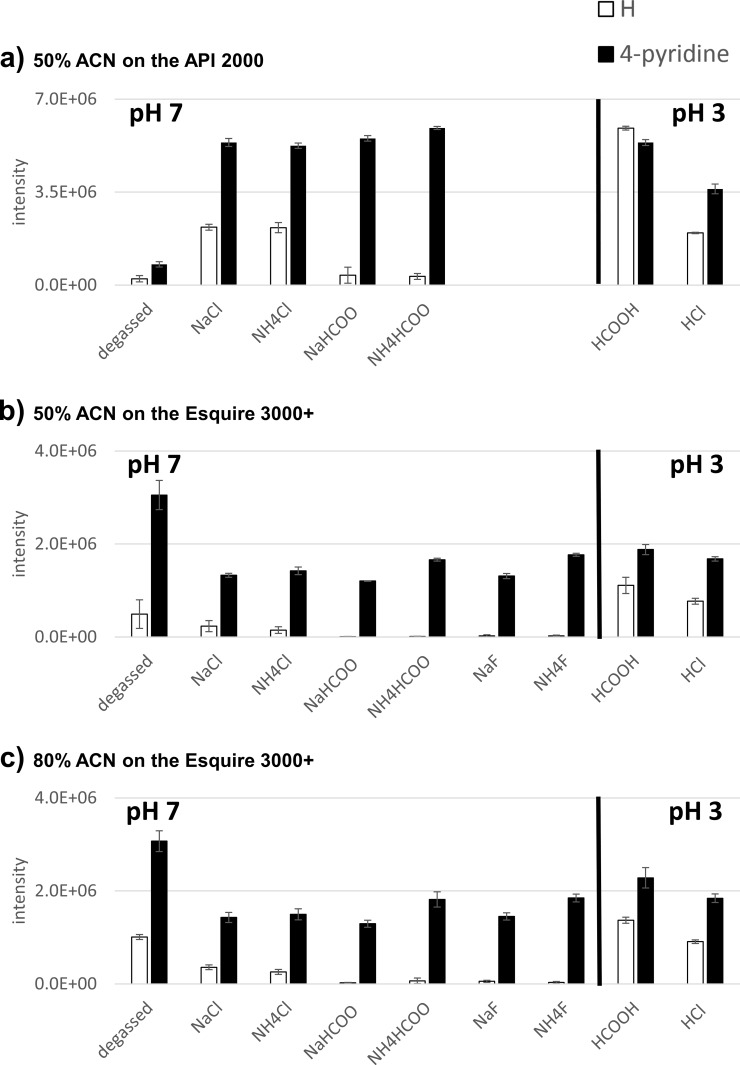
ESI response of aniline and 4-aminopyridine in presence of different, pH-modifying electrolytes. Analyses carried out a) by syringe pump infusion in 50% ACN on the API 2000, b) by sample flow injection in 50% ACN on the Esquire 3000+ and c) by sample flow injection in 80% ACN on the Esquire 3000+.

Unfortunately, pH adjustment is unavoidably associated with the addition of an electrolyte to the solution which ultimately competes with the corresponding target analyte for excess charge. Therefore, concentrations below 1 μM [36] or 10 μM [33] were suggested for additives to avoid any signal suppression effects. (*Note*: *this concentration is also dependent on the flow rate*.) We investigated potential signal suppression by pH modifiers for two representative substances, aniline as a moderately basic, comparably less polar analyte and 4-aminopyridine as a strongly basic, polar analyte. We followed two approaches, namely (i) adjusting the pH with two different types of pH modifier, formic acid as weak, volatile acid and HCl as strong, non-volatile acid, and (ii) adjusting the electrolyte concentration at 1 mM and pH 7 with combinations of different salts. The results are illustrated in Fig 1. (*Note: In the analyses of aminobenzonitrile with the sodium salt additives, we observed a highly abundant sodium adduct which is quite unusual for a compound that does not contain oxygen as a hetero atom. Since the presence of a sodium adduct complicates the data interpretation, this analyte is not discussed here. However, complete results are shown in S2 Fig*).

From the results, several conclusions can be drawn. In agreement with Ikonomou et al. [30], we clearly confirmed an ion suppression effect when comparing the response after addition of formic acid as volatile modifier to the response after addition of HCl as non-volatile modifier for pH adjustment. Though the molar amount of the weak acid formic acid as electrolyte added to obtain pH 3 was higher than the one required of the strong acid HCl, the response is lower in presence of HCl for both of the analytes, strikingly providing evidence that indeed the non-volatile acid exerts a stronger signal suppression. Though disadvantageous effects cannot completely be ruled out for formic acid as well it therefore still is the preferable pH modifier.

**Fig 2:**
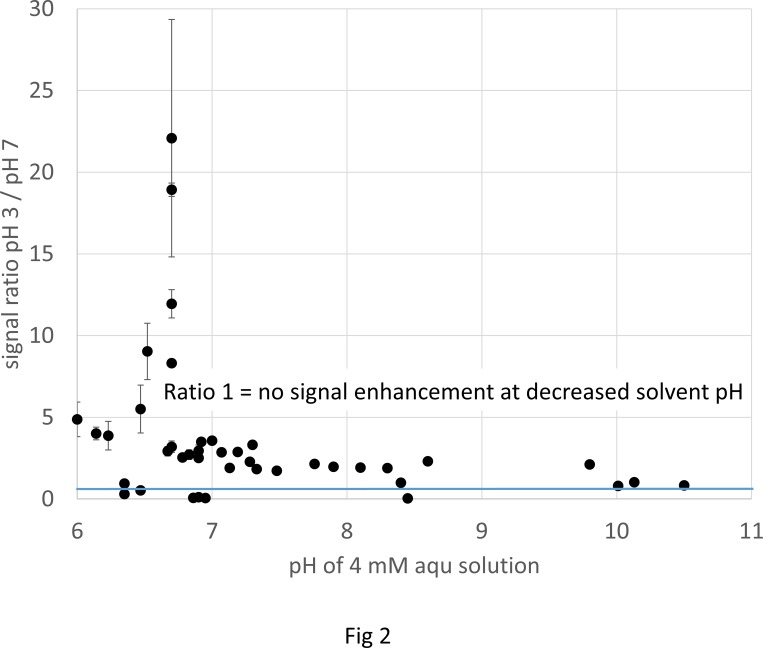
Response ratio of the ESI signal intensity at pH 3 and pH 7 in dependency on basicity. The response of every analyte in aqueous solution (pH 7) is compared to a solution adjusted to pH 3 by formic acid, analyzed for the whole set of analytes in 80% ACN on the API 2000.

All further comparisons suggest that strongly basic analytes are less prone to signal suppression than less basic ones, particularly at neutral pH. Very obvious, ESI-response of aniline as a less basic analyte is much more influenced by addition of electrolytes than the strongly basic 4-aminopyridine. This was confirmed using the whole dataset; Fig 2 presents the response ratios of signal intensities at pH 3 and pH 7 obtained from the full set of analytes in the original data set after flow injection by a syringe pump on the API 2000.

The weaker bases gave the highest response in acidic medium, where they are more extensively protonated, the strong bases were less enhanced by acidification. This tendency of increasing signal enhancement for less basic compounds by solvent acidification was observed and reported earlier [28], and also the signal response of strongly basic analytes was found independent on solvent pH [17,37]. In neutral solution, basic analytes already are completely protonated, so that the fraction of neutral species that can be further protonated by addition of acid additives is smaller compared to the less basic substances which therefore experience a larger increase in ion concentration by protonation at pH decrease. Therefore, the influence of the pH of the solvent on signal response might reflect the expected extent of protonation in solution.

Curiously however, when we repeated the ion suppression experiment on the second MS instrument, we found different results for the influence of solution pH (Fig 1B and 1C). Very clearly, we see a reversed behavior of the pure solutions as compared to all electrolyte-containing solutions: while on the API 2000 any electrolyte had a beneficial effect on signal response, on the Esquire 3000+ the electrolyte-free solutions exhibited the highest responses. This indicates that ion suppression of aniline on the Esquire 3000+ will occur upon addition of any electrolyte, while on the API 2000, the addition of electrolytes generally had a beneficial effect except for aniline with the two formates; eventually, aniline experiences a selective ion pairing effect with formic acid at neutral pH hampering the ion transfer to the gas phase. Concluding from that, the API 2000 would be the instrument with a higher tolerance against signal suppression by electrolytes.

The results observed on the API 2000 are in agreement to the findings of others [17,15,38] that for basic analytes it is not the solvent pH that matters but the availability of a suitable electrolyte. Eventually, the presence of an electrolyte increases the excess charge in the ESI droplet which in the following is occupied by the strongly basic analyte leading to a much higher availability of ions transferred to the gas phase. While on one hand, solvent electrolytes may shift the homeostasis of the analyte from the interior to the surface of the ESI droplet and produce a smaller droplet size [39] potentially leading to signal enhancement (as observed with the API, where additives that easily and completely dissociate produced signal enhancement of the analytes), on the other hand, they may be responsible for an impaired evaporation of the solvent potentially leading to signal suppression (as observed on the Esquire 3000+ where all electrolytes effected signal suppression) [36]. Concluding from our results, different instrumental configurations may determine which net effect may be finally observed.

Our findings further suggest that instrumental parameters and intrinsic configurations may be at least partially responsible for the controversial results on ESI influence parameter reported in the literature [21]. Instrumental parameters already have been described to influence ESI responsiveness, such as the source geometry, e.g. on-axis vs. off-axis [40], the source or heated capillary temperature [41] or nozzle-skimmer 0voltage (declustering potential) [42], which influenced the outcome of collision-induced dissociation. However, these effects were not described to cause instrument-related, specific *relative* differences as we observed here related to ion suppression. Schmidt et al. [43] found that the ion intensities after electrospray ionization were significantly enhanced and analyte fragmentation substantially reduced through desolvation by collisional activation if the pressure in the first pumping stage was increased. Yet, differential effects on compounds with different properties and with respect to signal suppression by electrolytes were not subject to investigations so far. For brevity, however, we discuss these effects with respect to the two instruments more detailed in S1 Appendix.

### Polarity and volatility are the most important molecular descriptors of ESI responsiveness of a compound following basicity

We always used at least two data sets for investigations on correlations of ESI signal response with available molecular descriptors, the original full dataset of 58 compounds analyzed on the API 2000 and the subset of 31 anilines reanalyzed on both instruments. S1 Table lists the linear correlation coefficients obtained between the assessed molecular descriptors and the signal intensity at pH 7, at pH 3 and the ratio of signal at pH 3 to the signal at pH 7. In general, stronger correlation coefficients obtained with Spearman’s correlation in most cases indicated either a potential, slightly unbalanced data distribution which could not be confirmed by visual inspection of the scatter plot matrix (S3 Fig, except vapor pressure) or nonlinear correlations.

Table 1 gives an overview about the correlations obtained for our data summarizing the molecular descriptors of basicity (pH of a 4 mM solution and pKa from Scifinder and ChemAxon, gas phase basicity), polarity (polar and solvent accessible surface area from ChemAxon, logD at pH 3 and pH 6, and logP from Scifinder and ChemAxon), molecular size (molecular mass, calculated molecular volume, molar volume from Scifinder) and volatility (boiling point and vapor pressure from Scifinder) in a way that one descriptor with a significant, linear correlation coefficient above 0.4 or below -0.4 would be enough to accept a correlation. (*Note: comprehensive values are available from S1 Table*.)

**Table 1:** Correlations between ESI response and molecular characteristics. The investigated molecular descriptors were summarized as descriptors of basicity (pKa, pH of 4 mM solution, gas phase basicity, proton affinity, substituent’s electronegativity, and polarizability), polarity (logD, logP, polar/nonpolar/solvent accessible surface area), size (molecular and molar volume, molar mass) and volatility (boiling point, vapor pressure, vaporization enthalpy). A significant linear correlation of signal intensity or ratio with one of the molecular descriptors of each group (basicity, polarity, size and volatility) with a coefficient >0.4 is denoted with “+”, a coefficient >-0.4 with “-“.

	all compounds	anilines API	anilines Esquire	*ortho*	*meta*	*para*
	*Signal intensity at pH 7 correlates with*					
signal pH 3	+	+	+		+	+
signal ratio	-	-	-	-		-
basicity	+	+	+	+	+	+
polarity	+	+	+	+	+	+
size						+
volatility						
	*Signal intensity at pH 3 correlates with*					
signal pH 7	+	+	+		+	+
signal ratio		-		+		
basicity	+	+			+	+
polarity	+	+			+	+
size			+	-		-
volatility		+				
	*Signal ratio pH 7/ pH 3 correlates with*					
signal pH 7	-	-	-	-		-
signal pH 3		-		+		
basicity		-		-*	+*	-
polarity		-	-	-		-
size						+
volatility	+	+	+	+		

*… gas phase basicity only

In the following, the correlations presented in Table 1 will be discussed in detail.

#### Basicity, polarity and volatility interact with solvent acidification for ESI responsiveness of compounds

Apart from all descriptors of relative basicity, namely pKa, the substituents orbital electronegativity, proton affinity and gas phase basicity, a slightly weaker, negative correlation was observed between ESI response and the molecular descriptors of compound polarity, namely logP, logD and polar/nonpolar surface area; there was almost no correlation of the polarity descriptors with the signal ratio of pH 3 / pH 7. The correlation between polarity and signal response was higher at pH 7 with both instruments indicating an interaction between compound polarity and solvent pH. In conclusion, polar analytes provide a higher relative ESI response at neutral pH, while non-polar analytes appeared to be less sensitive to solvent pH [37]. In contrast to Mandra et al. [23], who found the logD for pH 10–14 best correlated with ESI response at pH 7, the correlation of the response at both pH was strongest with logD at pH 3, potentially related to the fact that ESI in positive mode leads to acidification of the solvent by electrochemical oxidation of the ESI solvent. However, the influence of compound polarity on responsiveness in dependence on solution pH has not been extensively studied yet [28].

**Fig 3:**
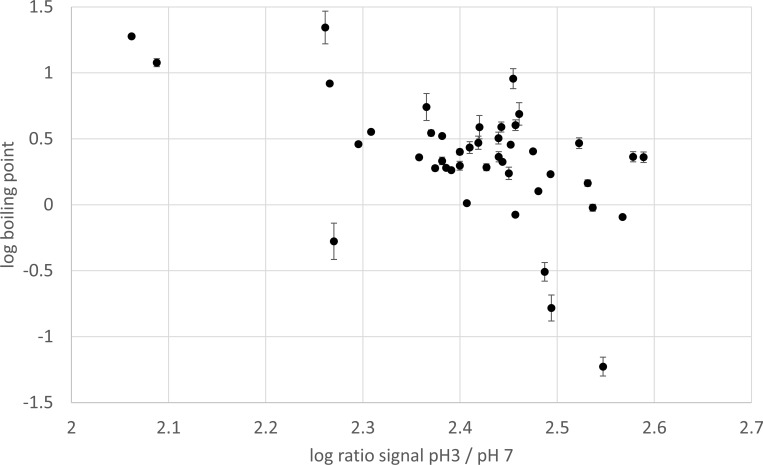
Signal enhancement by solvent acidification. Enhancement is more pronounced for compounds with lower boiling points. Response ratio pH 3 / pH 7 plotted over the boiling point, double logarithmic graph.

The same applies to the fact that the volatility of a compound exerted its influence particularly upon signal enhancement by acidification (response ratio, Fig 3). (*Note: vapor pressure was excluded from correlation analysis due to inappropriate data distribution, see S3 Fig*.)

Therefore, we suggest that the volatility of a compound is an additional advantage particularly in competition to a pH modifier, since this effect was independent from compound basicity itself (boiling point and basicity were not related). The interplay of these factors has not been stated and discussed in the literature yet, though an influence of volatility in general has been discussed earlier [44].

Finally, a very weak, nonlinear negative correlation was found between the molecular descriptors of the size of the molecule and signal intensity at pH 3. This curious finding may be related to the fact that our dataset was not particularly designed to assess this parameter since our substances were all quite similar in size compared to other studies using analytes much more different to each other when relating ESI responsiveness and molecular size [17–19,21–23].

#### For nonpolar compounds, ESI responsiveness is an interplay of solvent acidification and organic phase content

Interestingly, in the data set of the anilines only it was rather the signal enhancement by solvent acidification instead of the signal intensity at pH 3 that was related to logD at pH 3 but correlation strength attenuated again when enhancing the organic content of the solvent (80% ACN). It can be concluded that the nonpolar anilines will particularly benefit from acidification of the aqueous solvent only at low organic content; in agreement, at 80% ACN compound basicity and polarity correlated stronger with the absolute signal intensity than with the signal ratio. These observations are in well agreement with the understanding that increased organic phase content in the aqueous solvent facilitates the transition of ions into the gas phase due to decreasing (i) the surface tension and boiling point of the solvent and (ii) the polarity of the solvent reducing ion pairing effects with the analytes of interest [45–46]. Therefore, characteristics of ion formation (polarity and basicity) rather than ion transfer will determine ionization efficiency under these conditions.

Similar to our ion suppression experiment, different effects of molecular descriptors related to the instrumental configuration were reflected again within our data set (see S1 Appendix and S1 Table).

#### ESI responsiveness is related to the position of the substituent beyond basicity

The signal intensities of the *ortho*, *meta* and *para* substituted analytes showed the highest (linear) correlation coefficients to each other at pH 7 indicating that for a particular type of substituent the response pattern *ortho-meta-para* is conserved. Upon acidification however, i.e. at pH 3, the *meta* and *para* substituted analytes showed a more similar behavior to each other and to their signal intensity at pH 7, while the signal of *ortho* substituted ones at pH 3 was not related neither with the signal of *meta* and *para* substituted compounds at pH 7 or pH 3 nor with signal response ratio at both pH, where the coefficients had negative values (Table 2).

**Table 2:** Linear correlation coefficients between ESI responses of anilines with the same substituents at different positions, i.e. *ortho-meta-para*.

*Pearson’s correlation*	pH 7	pH 3	pH 3 / pH 7		
***ortho/meta***	0.69	-0.12	-0.41	*ortho* pH 7 vs. *meta* pH 3: 0.83	*ortho* pH 3 vs. *meta* pH 7:-0.49
***ortho/para***	0.64	-0.11	-0.34	*ortho* pH 7 vs. *para* pH 3: 0.77	*ortho* pH 3 vs. *para* pH 7: 0.38
***meta/para***	0.79	0.88	0.62	*meta* pH 7 vs. *para* pH 3: 0.91	*meta* pH 3 vs. *para* pH 7: 0.70

Moreover, the signal intensity of the *ortho* analytes at pH 3 was less dependent on compound basicity, negatively correlated with the electronegativity of the amino substituent, the compound polarizability, and molecular size (see also Table 1).

In conclusion, the *ortho*-substituted analytes were influenced by solvent acidification in a different way compared to *meta* and para. Thus, while for *meta* and *para* substituted analytes the signal at pH 3 rose with the relative nonpolar surface area (and logD at pH 3), for the “*ortho*-analytes” no such correlation was found. Instead and in contrast to the other anilines, the *ortho*-compounds showed a particularly strong correlation between signal enhancement after acidification and the vapor pressure. Eventually, the suggested H-sharing for the “*ortho*-analytes” (see S1 Appendix and [47–49]) may be responsible for the difference in comparison to *meta* and *par*a. The impact of position leads to a different “coherence” of the non/polar surface area, which would be highest for the *ortho*-position.

We checked which molecular descriptors differed the most for the *ortho*-substituted analytes and we found the electronegativity of the free amino group negatively correlated with the corresponding *meta*- and *para*-substituted analytes, meaning when the electronegativity of the amino group changed due to the presence of the second substituent, it changed in different direction for *meta* and *para* in comparison to the *ortho* position. In addition to that, the logP value was less similar for *ortho* to *meta* and *para* than *meta* and *para* to each other. Hence, these two descriptors can well be related to the H-sharing described in the literature [49].

## Conclusions

Our results confirm the well-established correlation of signal intensity with the compound basicity which is enhanced by the presence of electron-donating substituents ideally in *para* position. Interestingly, the response of compounds with substituents in *ortho* and *meta* position showed a much weaker correlation with basicity than *para* substituted ones what changed dramatically upon acidification, were the signal of *meta* and *para* substituents was still strongly correlated but for *ortho* analytes it was the electronegativity of the proton acceptor, the amino group, appearing to dominate the signal response behavior. A more detailed picture emerged from analysis of the influence of molecular descriptors on ESI responsiveness; the most prominent characteristic after basicity was the compound polarity expressed as logD at pH 3 that correlated consistently with signal intensity.

The signal enhancement after solvent acidification, i.e. the benefit of adding an acid pH modifier, was invers related to the signal intensity at pH 7 and compound polarity. Instead, the volatility of the analytes (boiling point and vapor pressure) becomes important at acid pH, so that the non-polar, more volatile compounds benefited the most from pH decrease. However, when decreasing the solvent pH, effects of ion suppression have to be considered.

Within this context and beyond, the most curious and interesting result of this study for ESI practitioners is the differential behavior of analytes on different types of instruments which is expected not to be restricted to the analytes selected here. The extent of ion suppression by solvent modifiers and the relative intensities of analytes obviously differ significantly at different instruments and conditions, and different molecular descriptors could become important for ESI-responsiveness. These findings have to be considered for method transfer, e.g. when comparing LODs achieved with different types of instruments and/or selecting the proper mobile phase for chromatography coupled to a particular instrument.

According to our results, a standardized ESI source design would be prerequisite for the development of standardized concepts for prediction of ESI responsiveness and improve the transferability of ESI-MS analytical methods from one instrument to the other.

## Supporting Information

S1 Appendix Detailed discussion related with the influence of solution basicity and instrumental configuration on ESI responsiveness. In this supporting information file, ESI responsiveness of the aromatic nitrogen-containing compounds is discussed in dependence on compound basicity and instrumental configuration of the two used instruments, i.e. API 2000 (AB Sciex) and Esquire 3000+ (Bruker).
(PDF)

S1 Fig Structural formulas of the compounds. The structural formulas of all analytes under investigation are presented, summarized in the groups of anilines, hydrazines, pyridines and pyrimidines.
(DOCX)

S2 Fig Ion suppression for aniline, 4-aminopyridine and 4-aminobenzontrile. Left: ESI-MS response in presence of 1 mM different, pH-modifying electrolytes. Analyses carried out a) by sample flow injection in 50% ACN on the Esquire 3000+ or b) by sample flow injection in 80% ACN on the Esquire 3000+. Bottom: mass spectrum of the sodium adduct of 4-aminobenzonitrile in 1 mM NaCl on the Esquire 3000+.
(PPTX)

S3 Fig Scatterplots for visual inspection of data distribution. All values are plotted against each other to assure proper distribution for establishment of linear correlations.
(PDF)

S1 Table Linear correlation coefficients between molecular descriptors and signal response at pH 7 and pH 3 and the signal response ratio at both pH. Data for the whole set of 58 analytes and the set of anilines analyzed on the two different instruments are presented.
(XLSX)

S2 Table Raw data of the experiments. (XLSX)
