# Supplementary material for: Electrospray Ionization Efficiency Is Dependent on Different Molecular Descriptors with Respect to Solvent pH and Instrumental Configuration
Source: PLoS One. 2016 Dec 1;11(12):e0167502. doi: 10.1371/journal.pone.0167502 (PMC5132301; doi:10.1371/journal.pone.0167502)
Supplement: S1 Appendix — In this supporting information file, ESI responsiveness of the aromatic nitrogen-containing compounds is discussed in dependence on compound basicity and instrumental configuration of the two used instruments, i.e. API 2000 (AB Sciex) and Esquire 3000+ (Bruker). (PDF) [file pone.0167502.s001.pdf]

S1 Appendix:

1. The fundamental relationship between ESI-MS response and solution basicity is reflected by ESI responsiveness of the aromatic nitrogen-containing compounds.

One of the most important compound characteristics known to determine the intensity of the MH+ signal in mass spectrometry after electrospray ionization is the extent of its protonation in solution, i.e. the solution basicity [1, 2]. The ability to attract a proton in solution is best described by the pKa of the respective compound that can be retrieved from public databases such as Scifinder and ChemAxon. In addition, we determined the extent of protonation and therefore the relative basicity of each analyte by assessment of the pH of a 4 mM aqueous solution. These values were plotted against the signal response of a 40 µM solution of each analyte, illustrated in Fig 1.

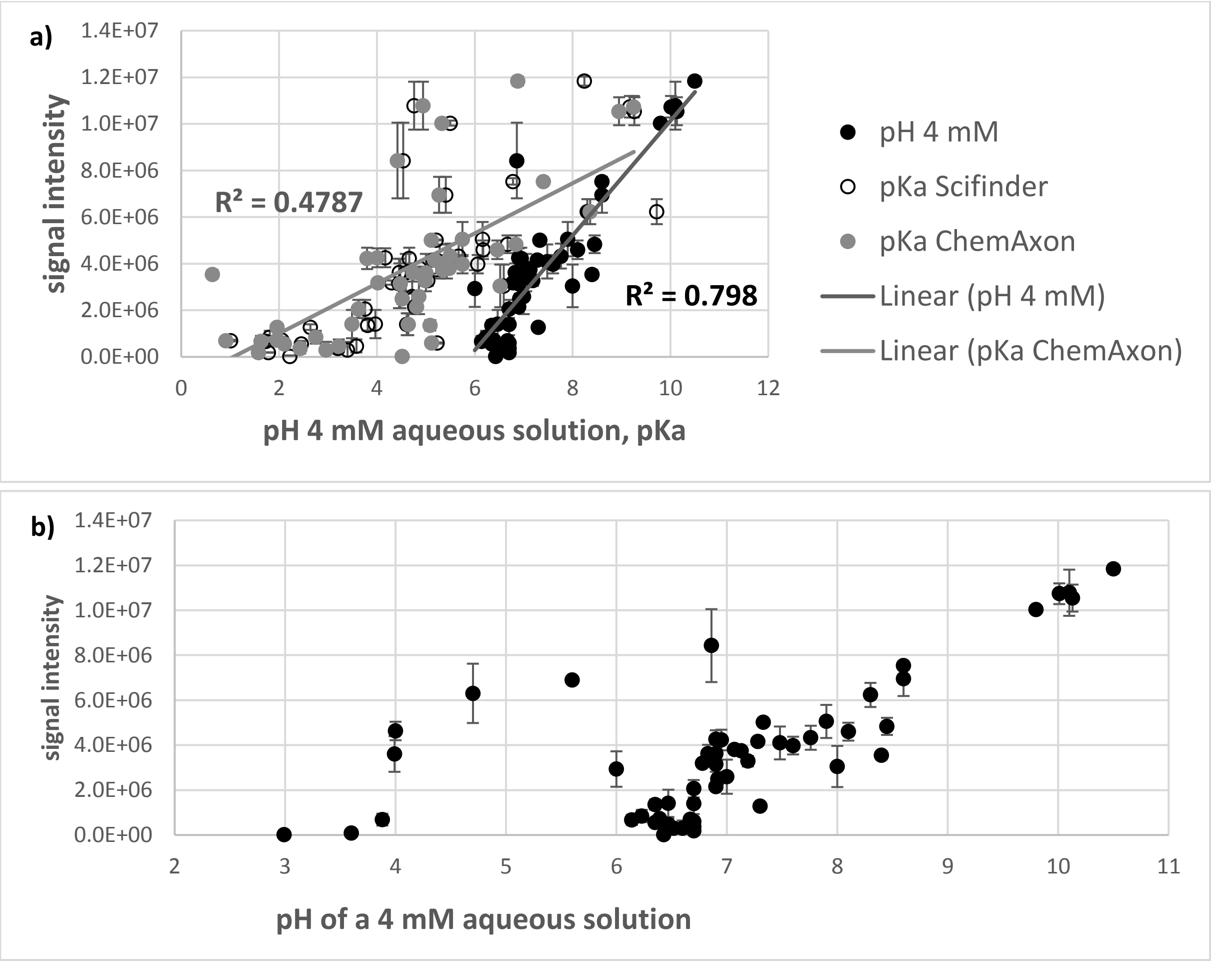

Fig 1. Basicity of the investigated compounds as determinant of ESI-MS sensitivity as signal response of a 40 µM solution. a) signal intensities of the peaks corresponding to the analytes' MH+ plotted over the pKa values [Scifinder, ChemAxon] and over relative basicity expressed as the pH of a 4 mM aqueous analyte solution. b) signal intensity over relative basicity expressed as pH of a 4 mM aqueous solution only for all analytes.

In confirmation of Ehrmann et al. [1], we found solution basicity and signal intensity linearly correlated above a relative basicity of pH 6 (Fig 1a). When including the less basic analytes (Fig 1b), R^2 was decreased and the relationship was better described by a polynomial relationship 3rd order. The correlation coefficient of response and relative basicity expressed in terms of the pH of a 4 mM aqueous solution was much stronger than the ones obtained with the mostly calculated data from public databases. Curiously, the aminobenzonitriles and the nitroanilines exhibited a relative solution basicity very different to the calculated values obtained from the databases; 2-nitroaniline in particular differed by a factor of >20. However, the

exclusion of these analytes from the data set would not change the linear correlation coefficients compared to the whole data set confirming the robustness of our findings.

With respect to the determination of solution basicity for a given analyte, we would like to emphasize that for aqueous ESI solvents degassing of the solution should be very carefully considered since we experienced gradual pH decrease earliest already during pH assessment with the electrode. This decrease is caused by carbon dioxide from air dissolving by time in the aqueous solvent and the related dissociation of carbonic acid which results in an approximate solvent pH of 5.6 after a while. Consequently, we always degassed our solvents right before the start of our experiment constantly feeding nitrogen through it. Notably, neutral pH without bubble formation in the solvent was best achieved by cooking the water and bubbling with nitrogen thereafter during analysis.

ESI-response is enhanced by electron-donating substituents

Solution basicity is mainly determined by inductive and mesomeric effects of substituents and their position, so that the response behavior of the analyzed compounds is expected to depend on the type and position of their substituents. Therefore, we investigated the influence of these characteristics in more detail. In particular, solution basicity of compounds was expected to be related to the electron withdrawing or donating character of a substituent. For aromatic amines, the lack of electrons in the benzene ring produced by electron-withdrawing substituents is the main reason for the extra planarity of the amine group, and it produces the shortening of the nitrogen–carbon bond distance, which is characteristic of the charged anilines. In all substituted anilines, the hydrogens of the amine group support different amounts of charge. Having the hydrogen next to the substituent results in the lowest electronic density; in *o*-diaminobenzene, on the other hand, the amine groups repel each other. There is a very close relationship between the strength of the substituent, the charge of the amine group, the ionization potential, and the basicity of the system [3]. The presence of a chemical substituent leads to a pronounced variation of the charge distribution in the molecule affecting structural and electronic parameters. Thus, compared to the structural conformation of aniline, considerable changes are expected from introducing substituents in *ortho, meta* or *para* position, including the flattening of the amine group [3]. Fig 2 illustrates the signal intensities for the subset of anilines in the order of their relative basicity.

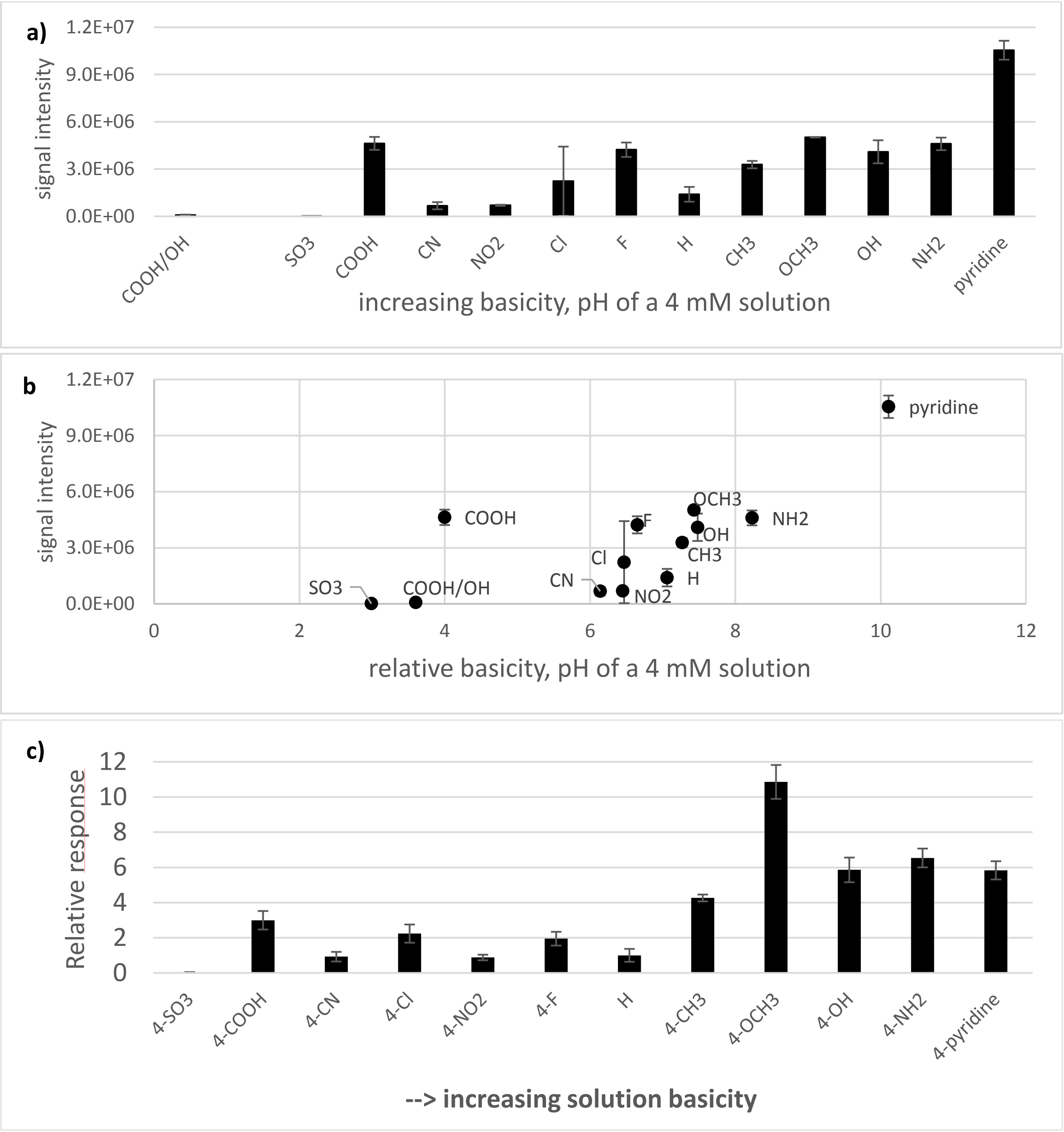

Fig 2. ESI responsiveness by solution basicity on different instruments. a) ESI response of all anilines with substituents at 4-position in 80% ACN on the API 2000, b) ESI response of all anilines with substituents at 4-position in 80% ACN on the API 2000 plotted over relative basicity, and c) ESI response of all anilines with substituents at 4-position in 50% ACN on the Esquire 3000 plus, normalized to the response of aniline.

Response behavior of the aromatic amines can be clearly differentiated by the electron withdrawing or donating character of the substituent with the exception of 4-aminobenzoic acid with an unexpectedly high response at both sets of analysis conditions (Fig 2 a and c). Hence, other parameters are expected to specifically influence ESI ionization of 4-aminobenzoic acid at both instruments and conditions. In addition, at 10 µM in 50% ACN on the Esquire 3000+, the methoxy substituent had an unexpectedly high response while in 80% ACN on the API 2000 4-aminopyridine has the highest intensity in agreement with its pKa. 4-aminopyridine has with Δ2 units the highest difference in relative basicity to its neighboring compound, comparable to the difference between aminoaniline and nitroaniline (illustrated in Fig 2b) or aniline and aminobenzoic acid, possibly accounting for this high difference in abundance at 80% ACN; all other relative basicities differ <0.5 between the compounds. Again, a linear relationship between pKa and ESI intensity could

be observed only above pH 6. Considering the type of substituent, all withdrawing substituents produced 4 mM solutions with pH values below 7 while all donating substituents in *para*-position had pH values above 7.

The signal response averaged over the *ortho*, *meta* and *para* position for each type of aniline substituent resembles in its pattern the one obtained for the *para*-subsituted compounds; the impact of the presence of electron-withdrawing and electron-donating substituents on signal intensity is striking (Fig 3a).

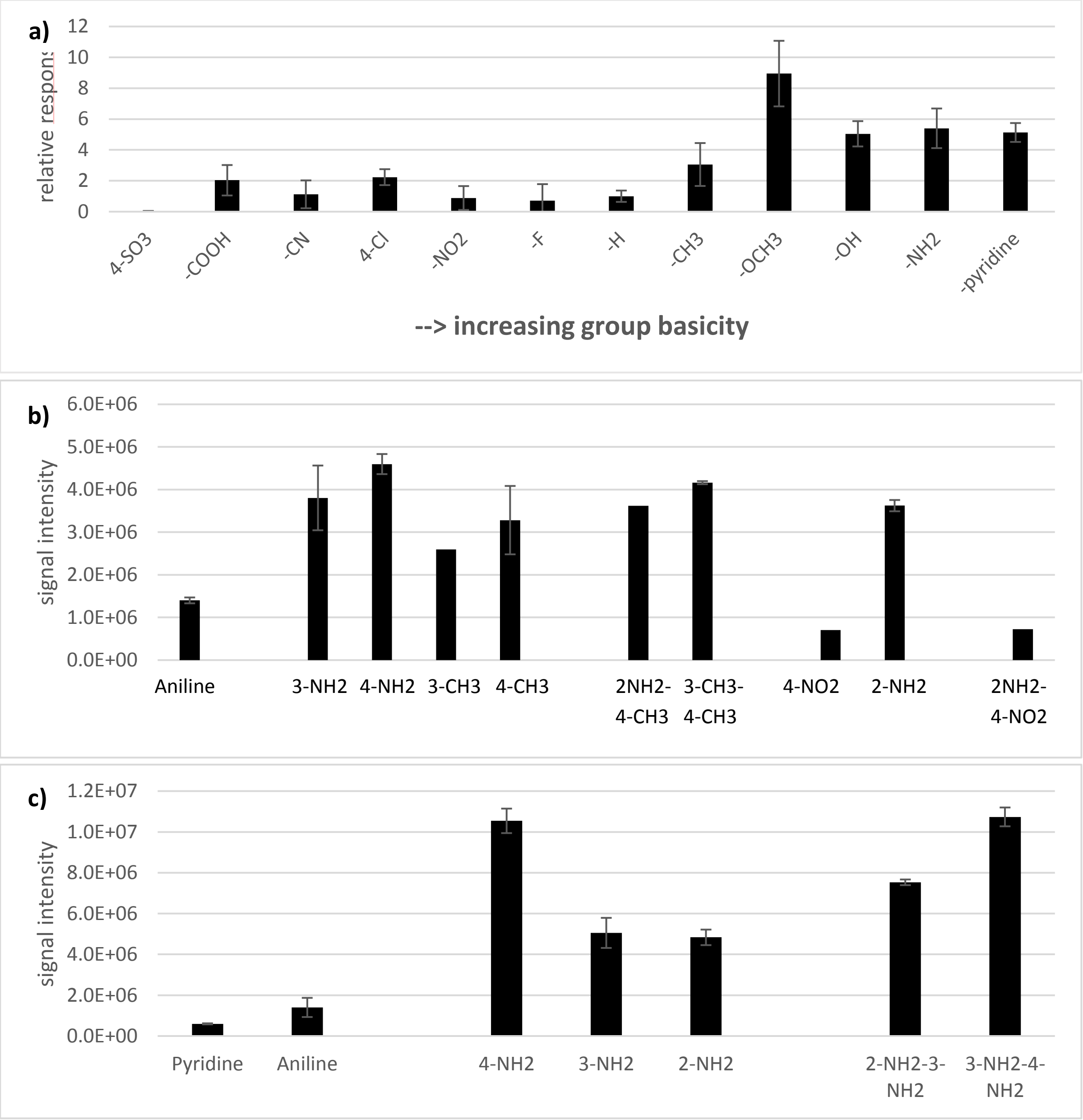

Fig 3. ESI responsiveness by enhanced substitution. a) relative signal response for the anilines with the Esquire 3000+, normalized to the response of aniline and averaged for the three positions of the corresponding subsitutent (*ortho*, *meta*, *para*) b) signal enhancement by increasing substitution grade for the anilines c) signal enhancement by increasing substitution grade for the pyridines

The influence of increasing substitution on signal intensity is illustrated in Fig 3 b and c. If at all, further signal enhancement was only achieved, if a more electron rich substituent was introduced; additional electron-withdrawing substituents resulted in a significant signal decrease. However, the extent of enhancement was much smaller comparing the introduction of the second to the third substituent. This finding is in agreement and confirms the notion that the selection of polyfunctional compounds with a limited range of ionization

efficiencies for studies on ESI responsiveness eventually hamper relating ionization efficiency to the molecular structure [4].

ESI-response is enhanced by introduction of substituents in *para*-position but decreased in *ortho*-position

Response depended clearly stronger on the type of substituent than on the position of the substituent. Thus while the responses by type of substituent (Fig 3) when averaged over the three positions of *ortho*, *meta* and *para* still were clearly different from each other, the standard deviations of the averaged responses of the substituted anilines over three positions overlapped largely (Fig 4).

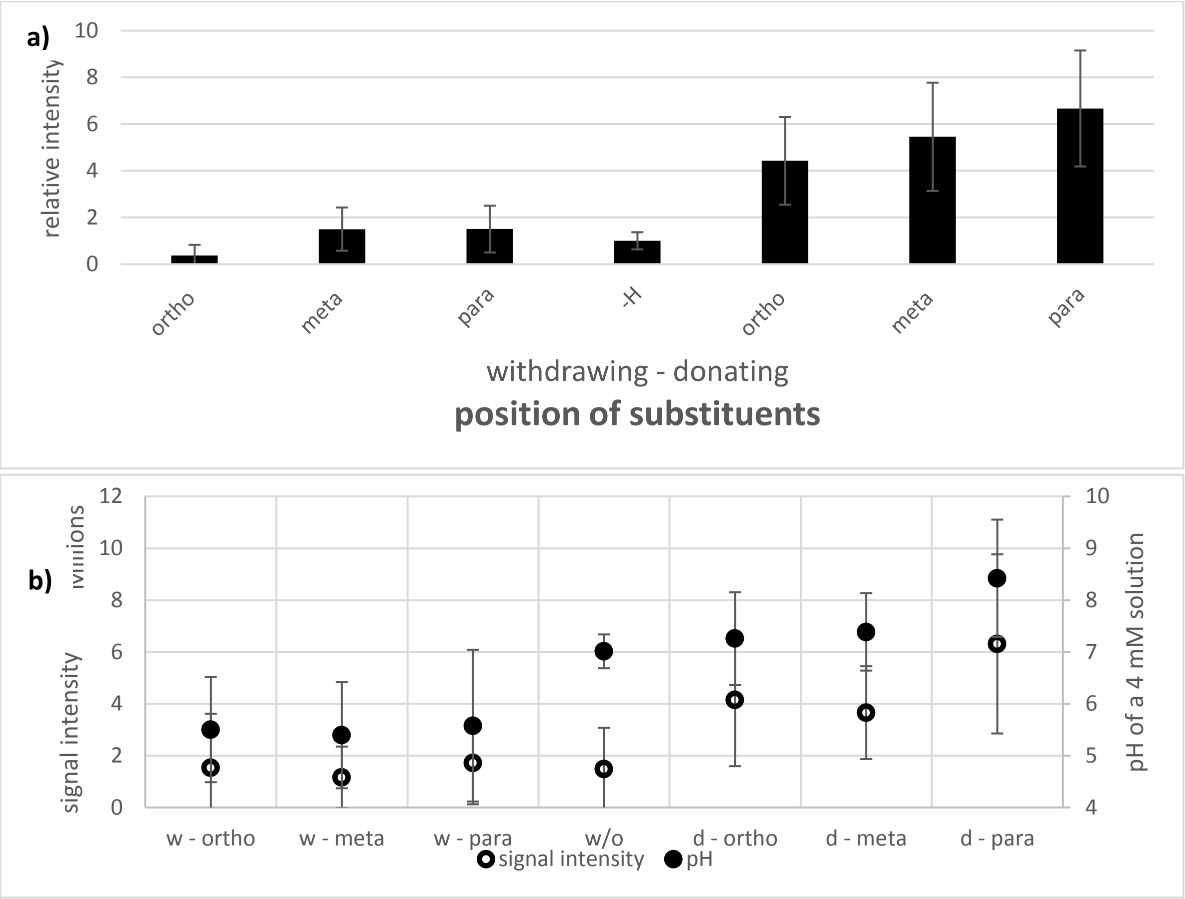

Fig 4. The effect of substituent position on ESI response. a) relative ESI response of all anilines in 50% ACN on the Esquire 3000+, normalized to the response of aniline and averaged over all positions, b) type of substituent (w-withdrawing, d-donating), position of the substituent, basicity and ESI-response of 44 different compounds in 80% ACN on the API 2000.

In conclusion, the general response pattern of electron-donating and electron-withdrawing substituents was on average conserved among the *ortho*, *meta* and *para* positions. The highest average response was obtained with the *para*-substituents, the lowest in *ortho* position. This is curious, because electron-donating mesomerism effects are strongest in *ortho* and *para*, electron-withdrawing at *meta*-position. Thus, the *para* and *ortho* carbon atoms of aniline have the largest negative charges compared to the other carbon atoms [5, 6]. The *ortho* carbon atoms show the greatest negative charge, attributed to a large mesomeric effect (1 M) owing to the non-bonding electrons of the amine group.

This charge distribution, however, is strongly affected by substituents. Vaschetto et al. [3] observed additional planarity of the amine group when an electron-withdrawing substituent group is added to the aniline. In particular, the atoms of *o*-nitroaniline all were (almost) in the same plane as the benzenoid ring, with a shortening of the bond length between the nitrogen and the benzene ring yielding a quinoid-like structure. In

anilines substituted with nitro and carboxy groups in *ortho* position containing an oxygen atom next to the hydrogen of the amine group, an intramolecular hydrogen bond is probably formed [7, 8]. This hydrogen bond is observed in both cases, it induces an additional planarity in the whole system, and in principle, should induce an increase in the electronic delocalization of the molecule thereby making it a weaker proton acceptor. The lack of electrons in the benzene ring produced by electron-withdrawing substituents is the main reason for the extra planarity of the amine group, and it produces the shortening of the nitrogen–carbon bond distance, which is in fact characteristic of the charged anilines. Also, carboxy, hydroxy, methoxy, trifluoromethyl and chlorine substituent groups in *ortho* position interact with the hydrogen of the amine group, although in these cases the interaction is weaker. Substituents in *meta* and *para* position on the other hand exhibit shorter distances to the neighboring H of the benzene ring and therefore, interactions with protons from solution may be stronger [3]. Therefore the low average responses for the *ortho*-substituted anilines for both types of substituents may be caused by H-sharing with the amino group decreasing the ability to attract a proton from solution.

We related the influence of electron donating and withdrawing effects dependent on the position of the substituent for 44 compounds featuring only two substituents to relative basicity and to signal intensity in comparison. Fig 4b presents the signal intensities averaged over all respective substituents compared to the corresponding averaged relative basicity. Again, signal intensity clearly follows relative basicity of the compounds. The strongest influence of the electron-donating effect was always in 4-position; here, the molecule can easier take up a more planar configuration to facilitate mesomerism for stabilization of the protonated molecule. In contrast, electron withdrawing effects are best compensated in *meta* position to the substituent. A comparison of ESI-responses in dependence on *meta* and *para* positioning of donating substituents in anilines, pyridines, pyrimidines and hydrazines is shown in Fig 5.

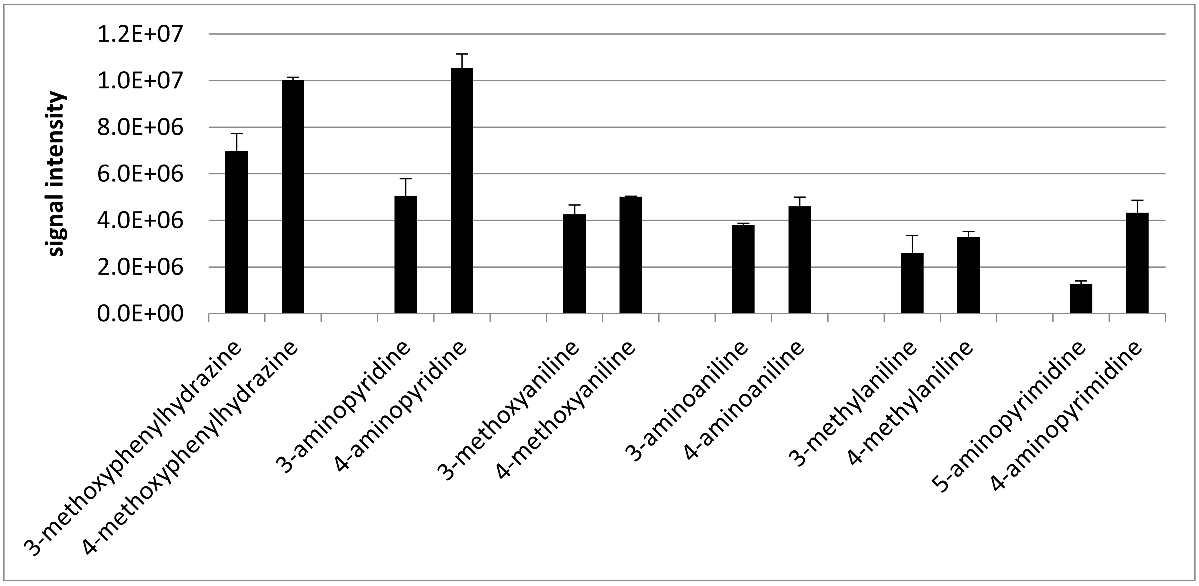

Fig 5. Effect of electron-donating substituents in *meta* and *para* position on ESI-MS response of in anilines, pyridines, pyrimidines and hydrazines.

Looking for effects of substituent positioning, ESI-response enhancement by relative basicity of 3- or 4-positioning of the second substituent was consistently reflected for electron-donating substituents. Very likely, the planar configuration for formation of mesomerism stabilizes the protonated molecular ion. Interestingly, response enhancement was least pronounced for the anilines. Generally, the formation of dipoles with increased electron density at one site would enhance the ability to attract and bind a proton. While lower signal intensities of protonated species in *ortho*-substituted anilines may be reasoned by H-sharing, a donating

substituent in 3-position is less likely to get involved with H-sharing. Instead, mesomeric resonance structures suggest the donating effect to be strongest at *ortho*- and *para*-position to the donating substituent and the amine function of the aniline itself. Thus, for donating substituents in *meta*-position to each other, the +M effects of the two substituents are not reinforcing each other as they would do in *para*-position.

Curiously, while for the electron-donating substituent group there is a clear succession for *ortho-meta-para* position in ESI-response, for the electron-withdrawing substituent group *meta* and *para* positions have a similar average intensity, sometimes even a higher intensity compared to the *para*-position (Fig 4 and 5). Anilines substituted with an electron-withdrawing group should be more stable when the substituent is placed in the *ortho* (or *para*) positions, whereas electron donor substituted anilines show a minimum in total energy when the group is bonded at the *meta* position [3] which could eventually be a reason for the observed positioning effect.

In conclusion, solution basicity and ESI-response are closely related with the interplay of electron-donating and withdrawing effects, i.e. the electron density of the investigated molecules and, therefore, cannot be separately assessed. However, other parameters may have the strongest impact in *meta*-position or absence of the second substituent where signal response shows the highest deviation from the relative basicity pattern (Fig 4b). ESI-response is determined by the same structural effects that account for the basicity of a compound.

2. Relative ESI responsiveness is determined by instrumental configuration.

Solvent pH decrease can have reversed effects on signal response with respect to the type of the instrument.

The assessment of solvent pH was accomplished adding a pH relevant electrolyte, namely formic acid. While on the API the addition of formic acid had a beneficial effect on signal response, a more complicated picture emerged from the results obtained with the Esquire for the set of anilines (Fig 6).

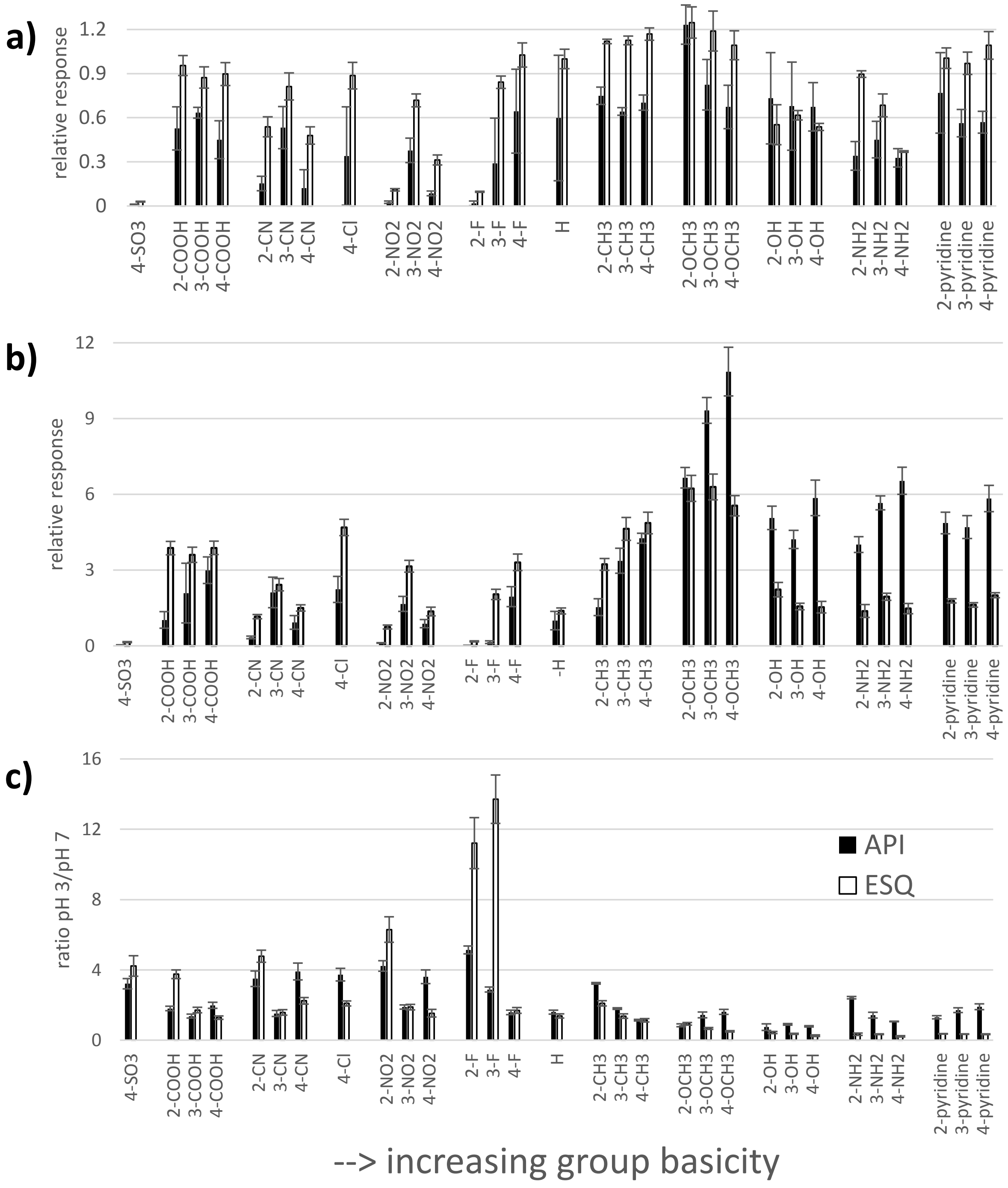

Fig 6. Comparison of ESI responsiveness normalized to the response of aniline of the corresponding data set. a) ESI response for the subset of anilines at pH 7 (black) and pH 3 (white) on the API 2000; b) ESI response for the subset of anilines at pH 7 (black) and pH 3 (white) on the Esquire 3000+; c) comparison of signal ratios pH 3 / pH 7 obtained in 50% ACN with the Esquire 3000+ and the API 2000.

On the API 2000, pH decrease consistently had a beneficial effect on almost all analytes (Fig 6a); moreover, the response pattern of *ortho-meta-para* was mainly preserved at the different pH. In contrast, on the Esquire 3000+ (Fig 6b), in particular the compounds with an electron-donating substituent exhibited even a higher intensity at pH 7 compared to pH 3, while the compounds with an electron-withdrawing second substituent mainly showed the expected behavior of signal enhancement upon addition of formic acid (Figs 6b and c). Eventually, solvent declustering effects, which might be stronger on the API 2000 considering the additionally applied curtain gas on this instrument, may play here a role as will be discussed in the next section.

Notably, the differences in ESI response were quite different: on the API 2000 (Fig 6a) the relative response to aniline was not higher than 1.2 while in the analyses from the Esquire the response of aniline was lower by factors, particularly when compared with analytes featuring an electron-donating second substituent such as the anisidines. In conclusion, the analyses carried out on the Esquire instrument showed a much more selective response pattern by compound basicity. By comparison of the ESI-response on both instruments in Fig 6 it can be observed that even the response pattern of *ortho-meta-para* between the two instruments changed in a few cases; while conserved on the API 2000, on the Esquire it was also different at different solvent pH. This emphasizes again that inherent instrumental parameter play indeed a different quantitative role.

Interestingly, while the median RSD of the samples analyzed at pH 3 was generally below 10%, this value increased considerably (<40%) for the samples analyzed at pH 7 (6% at pH 3 vs. 18% at pH 7, $p < 0.001$ for the whole set of analytes on the API 2000); hence, the addition of any electrolyte appeared to make the ionization process more reproducible which is in agreement with the observation of Olumee et al. [9].

Molecular descriptors determine signal response with respect to inherent instrumental configuration.

Correlations obtained for the subset of anilines with the Esquire 3000+ were generally weaker (in particular for solution basicity) but showed the same principal behavior when using the data obtained for the anilines measured on the API 2000 (S1 Table). Thus, solution basicity here was not correlated with the signal at pH 3 and signal enhancement upon solvent acidification. Interestingly, the negative correlation between the signal at pH 3 and the ratio of signal intensity at pH 3 /pH 7 was more pronounced when compared to the whole set of analytes providing evidence that with this instrument compound basicity is less important. An indeed crucial difference between the two instruments, however, is the emerging positive correlation of the nonpolar surface area, the solvent accessible molecular surface area and the molar volume with signal intensity at pH 3. This suggests that at acid solvent pH on the Esquire 3000+, the ionization efficiency is more sensitive to the processes of desolvation and interactions of ions in the gas phase than to compound solution basicity, a conclusion we also suggest within the context of the results of our ion suppression experiment (see related paragraph in the main article).

Moreover, compared to the Esquire, in the data set obtained for the anilines in 50% ACN with the API 2000 a stronger correlation regarding a negative influence of the size of the polar surface area and a positive influence of the (relative) size of nonpolar surface area on signal intensity at pH 3 could be established. This correlation was much weaker when the organic phase was increased to 80% ACN and suggests, that at acid pH the polarity of the molecular surface becomes important but in dependency on the amount of the organic or aqueous, respectively, phase. Also, the molecular mass correlated weakly with all three measures of ESI responsiveness on the API (negatively with the intensity and positively with the signal ratio) but not on the Esquire. Eventually, desolvation by solvent declustering through the nitrogen flow with the API 2000 is related with the weight since no correlation with molecular size was established on the other hand which might have hinted rather to processes of ion desorption. According to Schmidt et al. [10], hydrophilic (= polar) compounds have a low

affinity for the droplets surface and require a higher extent of solvent evaporation. They found that the ion intensities after electrospray ionization were significantly enhanced through desolvation by collisional activation if the pressure in the first pumping stage was increased. Thus, the presence of an additional curtain gas implemented on the API 2000 may be one reason related to the observed dependence of the signal on the molecular mass, as well as to the fact that the response obtained on this instrument was less prone to ion suppression upon addition of solvent electrolytes which is believed to be caused by impaired solvent evaporation [11] and, consequently, impaired solvent declustering. Within this context, we would like to draw the attention to the fact that one should carefully optimize the declustering potential with this type of instrument.

As a general conclusion, using correlation analysis we did not only find differences in ESI responsiveness related to compound characteristics, but by comparison of data sets obtained with different instruments we got hints that the ESI process as it runs on instruments of different vendors might not be as standardized as commonly assumed. Besides using different compounds for the investigations this may be at least part of the reason for different findings reported in the literature. Solution and gas phase basicity and chemistry, polarity (logP), the number of charge sites or different charge states in solution (pH), the susceptibility to oxidation/reduction, the tertiary structure and molecular size of the analyte (mainly for higher molecular weight compounds), vaporization energy or surface affinity all were suggested to influence the ESI process [11-20]. However, the findings were partially contradictory: thus for example, Zhou and Cook [21] found that signal intensities for caffeine and a very strong base (arginine) were independent on the pH of the solution, and Ehrmann et al. [1] did not find evidence for the importance of gas phase basicity.

The general importance of the fundamental parameters as there were compound basicity, polarity, and molecular size and weight, respectively, hold true to be factors indeed determining ESI responsiveness; their quantitative impact, however, is subject of interplay with other parameters such as solvent pH and instrumental configuration concluding from the results presented here. In fact, molecular descriptors such as the molecular weight, are not important for analysis with one instrument but may become important with the other hampering not only a consistent research on ESI mechanisms but also an efficient transfer of validated methods from one instrument to the other. Therefore, it may still be too early for such concepts as introduced by Leito et al. [12]; a standardization of the ESI sources prior to that might be a precondition for that.

3. **Supplementary note: thorough pH control is needed for investigating mechanisms of ESI responsiveness.**

Our results emphasize that solvent pH needs to be tightly controlled investigating relative ESI responsiveness of compounds to accurately decipher influential parameters and the mechanisms during the ionization process. In particular, constant solvent degassing should be understood as prerequisite in such experiments. In figure 7, we show the results obtained for the analysis of aniline and 4-aminopyridine for the two instruments used in this investigation.

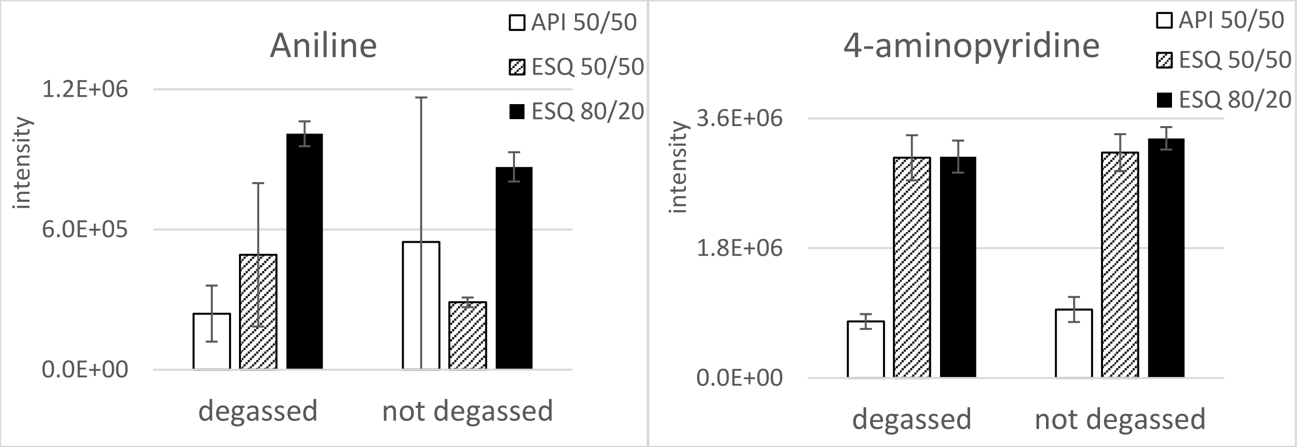

Fig 7. ESI responses of aniline and 4-aminopyridine in degassed and non-degassed aqueous solutions. a) ESI response for aniline on the API 2000 (white) and the Esquire 3000+ in 50% ACN (grey) and 80% ACN (black); b) ESI response for 4-aminopyridine on the API 2000 (white) and the Esquire 3000+ in 50% ACN (grey) and 80% ACN (black).

Though the basic 4-aminopyridine was hardly affected by degassing, a different ESI-response was observed for aniline that could potentially be related to the presence of carbonic acid as indicated by the higher solvent pH (5.7 for the non-degassed vs. 7.0 for degassed solution). Consistent with our results from the ion suppression experiment, on the API 2000 the potential presence of this electrolyte caused signal enhancement but signal suppression on the Esquire 3000+.

Interestingly, higher relative standard deviations (RSD) sometimes observed for ESI responses of analytes in non-degassed solvent, might be associated with the non-reproducible process of carbon dioxide dissolving in the distilled water and changing its pH heterogeneously at micro scale. In addition, analyses at pH 7 also required particularly long equilibration times between the measurements to prevent from any effects caused by the presence of electrolytes from previous measurements persisting in the instrument.

Moreover and in analogy to the presence of electrolytes in the solution, the RSDs of signal intensities appeared higher for solvents featuring a higher aqueous phase content which appeared to make the ionization process less reproducible.

4. References

1. Ehrmann BM, Henriksen T, Cech NB. Relative Importance of Basicity in the Gas Phase and in Solution for Determining Selectivity in Electrospray Ionization Mass Spectrometry. J Am Soc Mass Spectrom 2008; 19:719-28.
2. Mandra VJ, Kouskoura MG, Markopoulou CK. Using the partial least squares method to model the electrospray ionization response produced by small pharmaceutical molecules in positive mode. Rapid Commun Mass Spectrom 2015; 29:1661-75.
3. Vaschetto ME, Retamal BA, Monkman AP. Density functional studies of aniline and substituted anilines. J Mol Struct (Theochem) 1999; 468:209-21.
4. Oss M, Kruve Herodes K, Leito I. Electrospray Ionization Efficiency Scale of Organic Compounds. Anal Chem 2010; 82:2865-72.
5. Hehre WJ, Radom L, Pople JA. Molecular orbital theory of the electronic structure of organic compounds. XII. Conformations, stabilities, and charge distributions in monosubstituted benzenes. J Am Chem Soc 1972; 94(5):1496-1504.
6. Gorse A-D, Pesquer M. A theoretical study of aniline and some derivatives in their ground states. J Mol Struct (Theochem) 1993; 281:21-32.
7. Stewart R. The Proton: Applications to Organic Chemistry. London: Academic Press: 1985.
8. Nakagaki R, Aoyama I, Shimizu K, Akagi M. Hydrogen bonding of aromatic amines in hydroxylic solvents. Part 1. Absorption spectroscopy of substituted anilines and hydrogen bond-induced rehybridization of trivalent nitrogen atoms. J Phys Org Chem 1993; 6:261-72.
9. Olumee Z, Callahan JH, Vertes A. Droplet dynamics changes in electrostatic sprays of methanol-water mixtures. J Phys Chem A 1998; 102:9154-60.
10. Schmidt A, Bahr U, Karas M. Influence of pressure in the first pumping stage on analyte desolvation and fragmentation in nano-ESI MS. Anal Chem 2001; 73:6040-6.
11. Constantopoulos TL, Jackson GS, Enke CG. Effects of Salt Concentration on Analyte Response Using Electrospray Ionization Mass Spectrometry. J Am Soc Mass Spectrom 1999; 10:625-634.
12. Leito I, Herodes K, Huopolainen M, Virro K, Kunnapas A, Kruve A, Tanner R. Towards the electrospray ionization mass spectrometry ionization efficiency scale of organic compounds. Rapid Commun Mass Spectrom 2008; 22:379-84.
13. Gabelica V, De Pauw E, Karas M. Influence of the capillary temperature and the source pressure on the internal energy distribution of electrosprayed ions. Int J Mass Spectrom 2004; 231:189-95.
14. Enke CG. A Predictive Model for Matrix and Analyte Effects in Electrospray Ionization of Singly-Charged Ionic Analytes. Anal Chem 1997; 69:4885-93.
15. Voyksner RD. Combining Liquid Chromatography with Electrospray Mass Spectrometry. In: Cole RB, editor. Electrospray Ionization Mass Spectrometry, Fundamentals, Instrumentation & Applications. New York: Wiley; 1997. p. 323.
16. Van Berkel GJ, McLuckey SA, Glish GL. Electrochemical Origin of Radical Cations Observed in Electrospray Ionization Mass Spectra. Anal Chem 1992; 64:1586-93.
17. Cech NB, Enke CG. Practical implications of some recent studies in electrospray ionization fundamentals. Mass Spectrom Rev 2001; 20:362-87.
18. Kebarle P. A brief overview of the present status of the mechanisms involved in electrospray mass spectrometry. J Mass Spectrom 2000; 35:804-17.
19. Alymatiri CM, Kouskoura MG, Markopoulou CK. Decoding the signal response of steroids in electrospray ionization mode (ESI-MS). Anal Methods 2015; 7:10433-44.
20. Sigman ME, Armstrong PA, MacInnis JM, Williams MR. Equilibrium partitioning model applied to RDX-halide adduct formation in electrospray ionization mass spectrometry. Anal Chem 2005; 77:7434-41.
21. Zhou S, Cook KD. Protonation in electrospray mass spectrometry: wrong-way-round or right-way-round? J Am Soc Mass Spectrom 2000; 11:961-6.
