# Supplementary material for: Electrospray Ionization Efficiency Is Dependent on Different Molecular Descriptors with Respect to Solvent pH and Instrumental Configuration
Source: PLoS One. 2016 Dec 1;11(12):e0167502. doi: 10.1371/journal.pone.0167502 (PMC5132301; doi:10.1371/journal.pone.0167502)
Supplement: S1 Fig — The structural formulas of all analytes under investigation are presented, summarized in the groups of anilines, hydrazines, pyridines and pyrimidines. (DOCX) [file pone.0167502.s002.docx]

S1 Fig: Structural formulas of the compounds

Anilines

Hydrazines

Pyridines

Pyrimidines

Others
