# Supplementary material for: Electrospray Ionization Efficiency Is Dependent on Different Molecular Descriptors with Respect to Solvent pH and Instrumental Configuration
Source: PLoS One. 2016 Dec 1;11(12):e0167502. doi: 10.1371/journal.pone.0167502 (PMC5132301; doi:10.1371/journal.pone.0167502)
Supplement: S2 Fig — Left: ESI-MS response in presence of 1 mM different, pH-modifying electrolytes. Analyses carried out a) by sample flow injection in 50% ACN on the Esquire 3000+ or b) by sample flow injection in 80% ACN on the Esquire 3000+. Bottom: mass spectrum of the sodium adduct of 4-aminobenzonitrile in 1 mM NaCl on the Esquire 3000+. (PPTX) [file pone.0167502.s003.pptx]

## Slide 1
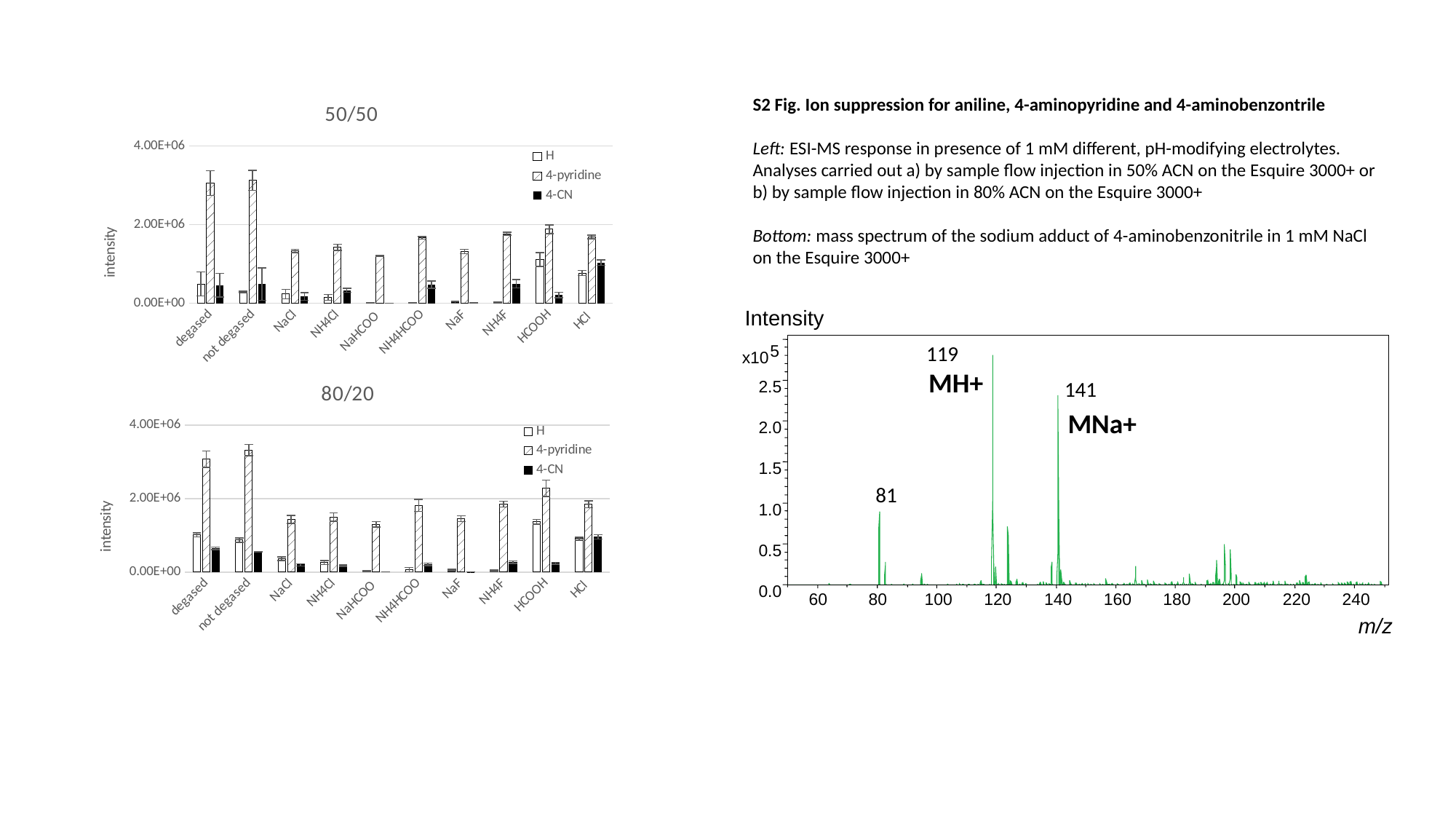

### Chart: 50/50
| Category | | | |
|---|---|---|---|
| degased | 491663.5 | 3053785.6666666665 | 457670.0 |
| not degased | 288307.5 | 3124944.3333333335 | 490365.0 |
| NaCl | 234385.66666666666 | 1327419.3333333333 | 174090.33333333334 |
| NH4Cl | 149539.33333333334 | 1422290.3333333333 | 331295.6666666667 |
| NaHCOO | 6432.0 | 1204479.3333333333 | 4053.0 |
| NH4HCOO | 10231.666666666666 | 1663238.0 | 471599.3333333333 |
| NaF | 25623.0 | 1312138.3333333333 | 6915.666666666667 |
| NH4F | 25356.333333333332 | 1767342.0 | 495644.0 |
| HCOOH | 1111167.0 | 1881895.0 | 209629.33333333334 |
| HCl | 769683.0 | 1681668.6666666667 | 1033801.6666666666 |S2 Fig. Ion suppression for aniline, 4-aminopyridine and 4-aminobenzontrile
Left: ESI-MS response in presence of 1 mM different, pH-modifying electrolytes. Analyses carried out a) by sample flow injection in 50% ACN on the Esquire 3000+ or b) by sample flow injection in 80% ACN on the Esquire 3000+
Bottom: mass spectrum of the sodium adduct of 4-aminobenzonitrile in 1 mM NaCl on the Esquire 3000+
Intensity
5
119
x10
2.5
141
2.0
1.5
81
1.0
0.5
0.0
60
80
100
120
140
160
180
200
220
240
m/z
MH+
MNa+
### Chart: 80/20
| Category | | | |
|---|---|---|---|
| degased | 1009757.0 | 3070444.3333333335 | 644561.3333333334 |
| not degased | 868169.0 | 3323622.6666666665 | 542627.0 |
| NaCl | 358431.6666666667 | 1429264.6666666667 | 197029.33333333334 |
| NH4Cl | 259294.0 | 1495688.6666666667 | 177466.33333333334 |
| NaHCOO | 24672.0 | 1295499.3333333333 | 4686.333333333333 |
| NH4HCOO | 67099.33333333333 | 1815796.0 | 208931.66666666666 |
| NaF | 55423.0 | 1450702.0 | 4351.0 |
| NH4F | 32376.0 | 1848045.3333333333 | 269190.3333333333 |
| HCOOH | 1369734.0 | 2280676.0 | 240706.0 |
| HCl | 908658.6666666666 | 1842752.3333333333 | 957124.0 |
