# Supplementary figures and images for: Electrospray Ionization Efficiency Is Dependent on Different Molecular Descriptors with Respect to Solvent pH and Instrumental Configuration
Source: PLoS One. 2016 Dec 1;11(12):e0167502. doi: 10.1371/journal.pone.0167502 (PMC5132301; doi:10.1371/journal.pone.0167502)

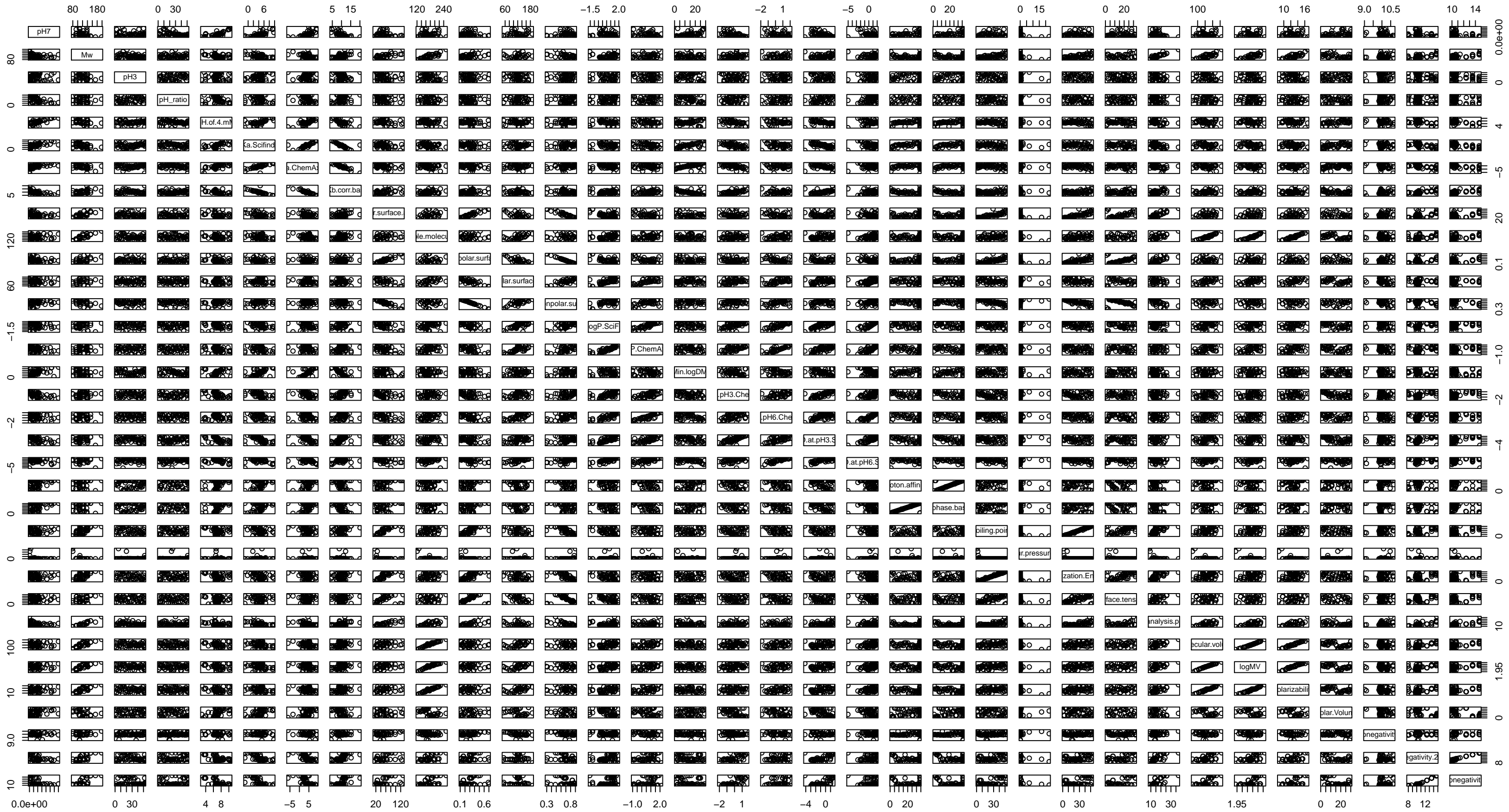

Supplement: S3 Fig — All values are plotted against each other to assure proper distribution for establishment of linear correlations. (PDF) [file pone.0167502.s004.pdf]
